# Identification of Potential Ferroptosis Biomarkers in Multiple Myeloma via WGCNA and Experiments

**DOI:** 10.1002/cnr2.70446

**Published:** 2025-12-29

**Authors:** Yifan Wang, Xing Xie, Mengyuan Gu, Yanting Zheng, Jing Wu, Qicai Wang, Zhian Ling, Ruolin Li

**Affiliations:** ^1^ Department of Clinical Laboratory First Affiliated Hospital of Guangxi Medical University, Key Laboratory of Clinical Laboratory Medicine of Guangxi Department of Education Nanning Guangxi China; ^2^ Department of Scientific Research First Affiliated Hospital of Guangxi Medical University Nanning Guangxi China; ^3^ Department of Emergency Second Affiliated Hospital of Guangxi Medical University Nanning Guangxi China

**Keywords:** BCAT2, CDKN1A, ferroptosis, multiple myeloma, WGCNA

## Abstract

**Introduction:**

Multiple myeloma is a common malignant tumor of the hematologic system, and genetic alterations play a crucial role in its occurrence and development. Ferroptosis is an oxidative and iron‐dependent programmed cell death, which has a strong correlation with tumor development. This study aimed to identify potential diagnostic ferroptosis‐related genes of MM.

**Methods:**

MM datasets were screened from the GEO database using publicly available transcriptomic data. Ferroptosis‐related hub genes were identified through enrichment analysis, WGCNA, and machine learning algorithms. ROC curves, boxplots, RT‐qPCR, and ELISA were conducted to validate the expression levels of these hub genes.

**Results:**

A total of 178 ferroptosis‐related DEGs were identified, including 114 up‐regulated genes and 64 down‐regulated genes. Enrichment analysis indicated that the DEGs were primarily associated with stress, autophagy, and metabolism. According to the WGCNA, the brown module has the highest correlation with clinical symptoms, containing 1141 DEGs. Combining with the identified ferroptosis‐related DEGs, two hub genes of CDKN1A and BCAT2 were identified by multiple bioinformatics techniques of LASSO, SVM, and Random Forest. ROC curves demonstrated strong diagnostic values in CDKN1A (test set, AUC = 0.881; validation set, AUC = 0.705) and *BCAT2* (test set, AUC = 0.808; validation set, AUC = 0.756). RT‐qPCR confirmed that the mRNA expression levels of CDKN1A and BCAT2 in three MM cells (RPMI 8226, WT‐U266, and LP‐1) were both significantly higher than the control HMy2.CIR cell line (*p* < 0.05). ELISA quantification revealed significantly elevated relative expression of CDKN1A protein in the MM cohort compared to healthy controls (*p* < 0.01), but the protein expression of BCAT2 exhibited comparable levels *(p* > 0.05).

**Conclusion:**

Our results suggest that CDKN1A and BCAT2 are potential ferroptosis‐related biomarkers for MM. This may help understand the molecular mechanisms and therapeutic strategies of MM.

**Abbreviations**
AML: acute myeloid leukemia
AUC: area under the curve
BCAA: branched‐chain amino acid
BCL‐2: B‐cell lymphoma‐2
DEGs: differentially expressed genes
DLBCL: diffuse large B‐cell lymphoma
ELISA: enzyme linked immunosorbent assay
FRGs: ferroptosis‐related genes
GEO: gene expression omnibus
GO: gene ontology
GPX4: glutathione peroxidase 4
GSEA: gene set enrichment analysis
GSH: glutathione
HMGB1: high mobility group box 1
KEGG: Kyoto encyclopedia of genes and genomes
MGUS: monoclonal gammopathy of undetermined significance
MM: multiple myeloma
NCBI: National Center for Biotechnology Information
PanIN: pancreatic intraepithelial neoplasia
PDAC: pancreatic ductal adenocarcinoma
ROC: receiver operating characteristic curves
ROS: reactive oxygen species
RT‐qPCR: quantitative real time polymerase chain reaction
SVM: support vector machine
TOM: topological overlap matrix
WGCNA: weighted gene coexpression network analysis

## 1 Introduction

Multiple myeloma (MM) is a malignant neoplastic disease characterized by abnormal proliferation of clonal plasma cells and secreting large amounts of monoclonal immunoglobulins [1]. The precise cause of multiple myeloma is still unclear, but it is widely believed to be related to genetic mutations [2]. Cytogenetic abnormalities and molecular alterations such as hyperdiploid mutation can influence the progression, treatment response, and prognosis of MM [3]. For patients with certain specific genetic markers, such as those exhibiting the genetic marker t (11;14), the oral B‐cell lymphoma‐2 inhibitor venetoclax has been shown as an effective treatment for MM [4]. Therefore, exploring potential biomarkers, diagnostic genes, and pathogenesis of MM holds significant value.

Ferroptosis is a mode of programmed cell death defined by buildup of iron within cells and lipid peroxidation, ultimately leading to oxidative stress and ensuing cell death [5]. This type of programmed cell death was first discovered and reported by Scott J. Dixon in 2012 [6]. Many studies have demonstrated that ferroptosis is inextricably bound up with the development of MM, and plasma cells have the capacity to undergo ferroptosis [7]. Abundance of SLC7A11 dictated the susceptibility of MM cells to erastin‐induced ferroptosis; genetic knockdown of SLC7A11 significantly suppressed MM cell proliferation and triggered characteristic ferroptotic events, including lipid peroxidation accumulation and glutathione depletion [8]. Mechanistically, bortezomib elevates intracellular free Fe^2+^ by enhancing NCOA4‐mediated ferritinophagy and synergizes with RSL‐3 by increasing ferroptosis in MM cells [9]. So ferroptosis‐related genes (FRGs) may have the potential to help us predict, diagnose or treat MM.

With the swift advancement of molecular diagnostics, more and more researchers are beginning to conduct studies based on bioinformatics data analysis to winnow characteristic biomarkers [10]. Among them, the Gene Expression Omnibus (GEO) database (https://www.ncbi.nlm.nih.gov/gds) of the National Center for Biotechnology Information (NCBI) provides scholars with a vast amount of gene expression profiles for various diseases. Through the analysis of relevant data on GEO, Zhai M. et al. found that the KIF22 is an important research target for exploring the pathogenesis, diagnosis, and treatment of multiple myeloma [11].

Peter Langfelder and Steve Horvath developed the Weighted Gene Co‐expression Network Analysis (WGCNA) in 2008 [12]. And it can be referred to as a method for gene screening and exploration.

In this study, we leveraged WGCNA, LASSO regression, SVM (Support Vector Machine), and Random Forest algorithms to screen ferroptosis‐related differentially expressed genes (DEGs) in MM. Subsequently, we used visual models, such as boxplots and receiver operating characteristic curves (ROC), to analyze their value. In addition, RT‐qPCR and ELISA experiments helped us verify the relative expression levels of selected hub genes in MM cells and patient samples. Our results indicated FRGs may influence the gene pathways and functional changes associated with MM, which provide new directions and insights for MM research.

## 2 Methods

### 2.1 Data Collection

Expression profiles publicly available on the GEO database (https://www.ncbi.nlm.nih.gov/geo/) prior to May 2024 were searched and collected. Expression profiles were retained only if they fulfilled both criteria: (1) a minimum of 30 subjects and (2) including both normal controls and patient data. After screening, only three eligible expression profiles (GSE5900, GSE6477, and GSE146649) were downloaded.

GSE5900 and GSE146649 were based on GPL570 platform as the test dataset [13, 14], including 32 cases of healthy donors and 87 cases of patients. These 87 patients include 44 samples of monoclonal gammopathy of undetermined significance (MGUS), 12 samples of smoldering myeloma, and 31 MM samples. For this study, we included 87 MM samples and 32 control samples as research objects. Then, we downloaded GSE6477 based on GPL96 platform as the validation dataset [15]. It includes 15 normal donors and 147 MM samples.

FRGs were downloaded from the FerrDb (http://www.zhounan.org/ferrdb/current/) [16]. This gene list contained 728 ferroptosis‐related genes, including 369 ferroptosis driver genes, 348 ferroptosis suppressor genes, and 11 ferroptosis marker genes.

### 2.2 Data Preprocessing and Ferroptosis‐Related DEGs Identificating

Limma [17] and Sva [18] R packages were applied to merge dataset GSE5900 and GSE146649. Differential expression analysis of ferroptosis‐related genes was performed using the R package “limma” with selection standards of |log FC| > 2 and *p*‐value < 0.05. Use the “heatmap” and “ggplot2” packages to visualize heatmaps and volcano plots.

### 2.3 Enrichment Analysis

We used the R package “cluster Profiler” for gene ontology (GO) term analysis and Kyoto Encyclopedia of Genes and Genomes (KEGG) pathway analysis to identify the functional roles of the ferroptosis‐related DEGs [19]. At the same time, we selected c2.cp.kegg_legacy.v2023.2.Hs.symbols.gmt as the reference gene set. After that, gene set enrichment analysis (GSEA) was conducted. Set *p*‐value less than 0.05 as the standard for significant enrichment.

### 2.4 Coexpression Network Analysis and Module Selection

We constructed gene coexpression networks of the merged gene expression profiles of GSE5900 and GSE146649 by using the WGCNA package in R [12]. We used the “WGCNA” package to explore the modules of highly correlated genes among samples for relating modules to outer sample traits. A scale‐free network was constructed. Next, we converted it into a topological overlap matrix (TOM) and the corresponding dissimilarity (1‐TOM). Later, we implemented a clustering dendrogram of the 1‐TOM matrix and classified at least 60 similar genes into different gene co‐expression modules. We set the abline as 0.25 to merge similar modules on the clustering tree. After relevance analysis, we integrated the information and identified the most pertinent module of MM.

### 2.5 Hub Genes Screening by Machine Learning

Use a Venn diagram to intersect the most relevant module with ferroptosis DEGs [20], and the results were intersection genes. LASSO, SVM, and Random Forest were used to analyze the intersection genes to screen significant variables. R package “glmnet” was used for performing LASSO regression analysis [21]. SVM selects the most important subset of features by recursively training an SVM model and eliminating the features with the smallest weights. In our study, package “e1071” was used to conduct SVM analysis [22]. Random Forest is an ensemble learning method that makes predictions and classifications by constructing multiple decision trees [23]. Package “randomForest” and “ggplot2” were used to run Random Forest analysis. The obtained intersecting genes of the three algorithms were identified as hub genes.

### 2.6 Verification and Analysis of Hub Genes

The expression differences of hub genes between control and treatment groups were evaluated by using boxplot. Also, ROC curves were utilized to verify the dependability of hub genes in validation dataset [24].

### 2.7 Quantitative Real Time Polymerase Chain Reaction (RT‐qPCR)

Use Trizol (Takara, Japan) to extract total RNA from RPMI 8226, WT‐U266, LP‐1 and HMy2.CIR cells (Pricella, China). The NanoDrop One (Thermo Scientific, America) instrument was used to determine the purity and concentration of extracted RNA. PrimeScript RT reagent kit (Takara, Japan) was used to remove Genomic DNA and reverse transcribe RNA into cDNA in our RNA samples. RT‐qPCR was performed on BCAT2, CDKN1A and human β‐actin using TB Green Premix Ex Taq II (Takara, Japan). Human β‐actin was set as the internal reference gene. The primers were synthesized by Sangon Biotech (China). The sequences of our primers are as follows:

BCAT2 forward 5′‐AAATGGGCCTGAGCTGATCC‐3′; BCAT2 reverse 5‐GAGTCATTGGTAGGGAGGCG‐3′; CDKN1A forward 5′‐AGCAGAGGAAGACCATGTGGA‐3′；CDKN1A reverse 5‐AATCTGTCATGCTGGTCTGCC‐3′; β‐actin forward 5′‐CATGTACGTTGCTATCCAGGC‐3′; β‐actin reverse 5‐CTCCTTAATGTCACGCACGAT‐3′.

### 2.8 Patient Collection

Serum samples were collected from 30 newly diagnosed MM patients of the Department of Hematology at the First Affiliated Hospital of Guangxi Medical University from April 2024 to April 2025. And 30 healthy controls were from Physical Examination Center. All steps included in this experiment met the principles in the Declaration of Helsinki. MM patients with any of the following issues will be excluded: (1) HIV, hepatitis B virus, hepatitis C virus, or other viral infections (2) neoplastic disease; and (3) other hematological diseases. This research recruited 30 healthy individuals as a control group without any previous hematopathy, inflammation, or tumor history.

### 2.9 Enzyme Linked Immunosorbent Assay (ELISA)

BCAT2 and CDKN1A protein levels of normal and MM serum samples were measured by Human BCAT2 ELISA kit (cat. no. F0895‐HB, Fankew, China) and Human CDKN1A ELISA Kit (cat. no. F0886‐HB, Fankew, China). The steps were performed according to the manufacturer's instructions. Use Multiskan GO Full Wavelength Enzyme Labeler (Thermo Fisher, China) to measure the OD of the test wells at 450 nm. According to the measurable range of the specification, exclude the failed data.

## 3 Results

### 3.1 Identification of DEGs in MM

**FIGURE 1:**
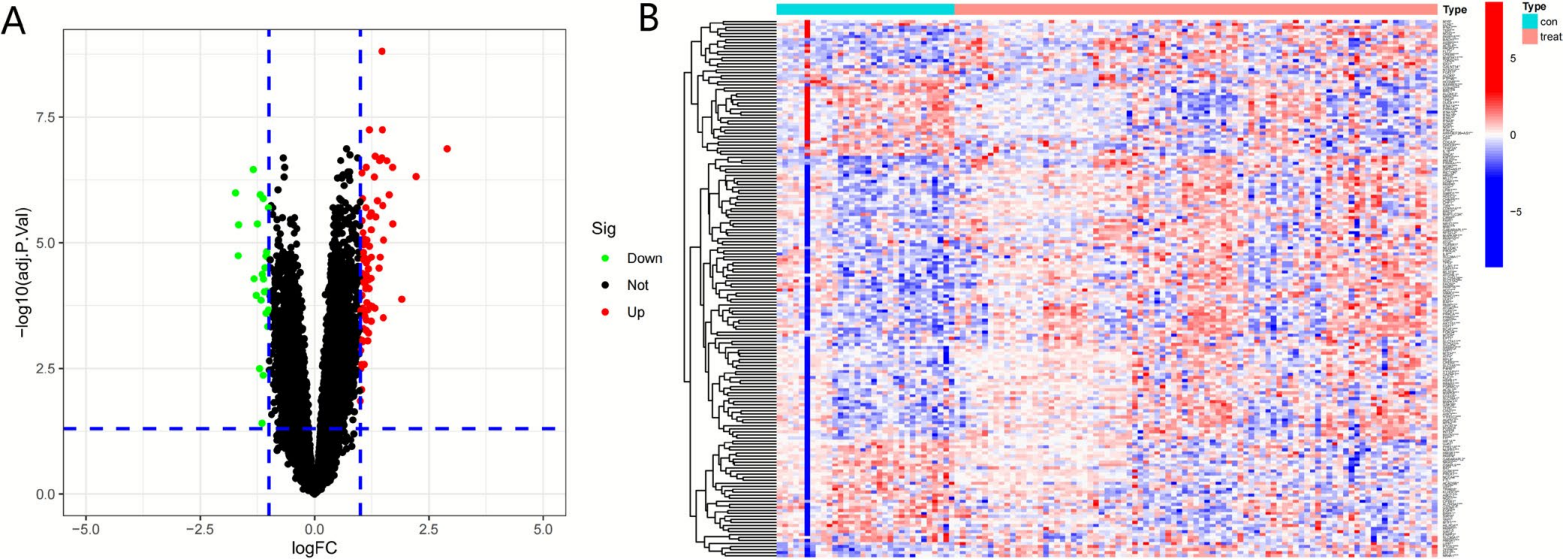
Analysis of DEGs in MM. (A) Volcano plot of DEGs. The red dots represent the up‐regulated genes and the green dots represent the downregulated genes, while black dots represent nonsignificant genes. (B) Heatmap of the top 50 differentially expressed genes.

After differential expression analysis of FRGs, we obtained a total of 114 up‐regulated genes and 64 down‐regulated genes. Our results were shown as volcano plot (Figure 1A) and heatmaps (Figure 1B).

### 3.2 Enrichment Analysis

**FIGURE 2:**
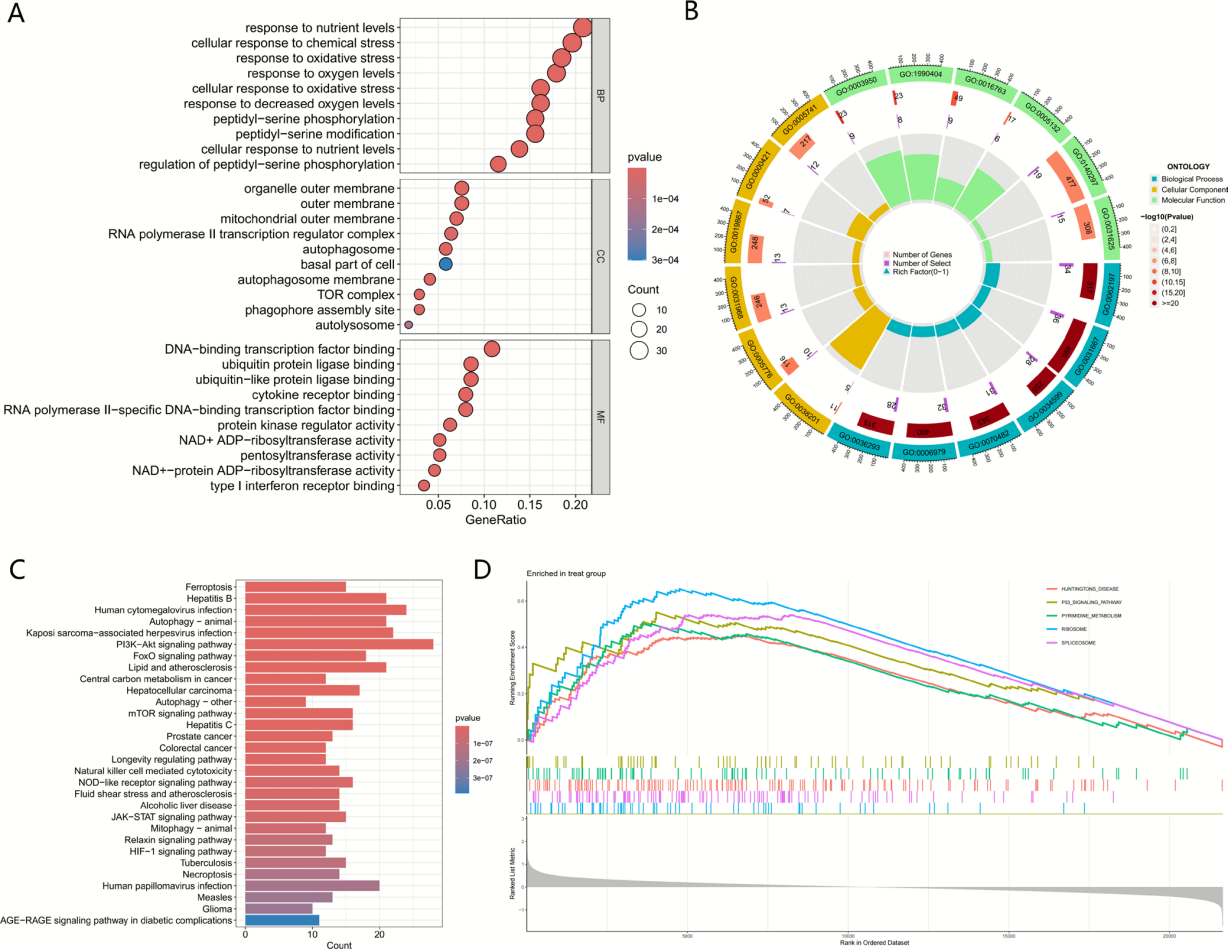
Functional enrichment analysis of DEGs. (A) GO enrichment analysis (BP, biological process; CC, cellular component; MF, molecular function). (B) Circleplot of GO enrichment analysis. (C) KEGG pathway enrichment analysis. (D) GSEA results in the treat group (MM group).

To understand the potential functions and metabolic pathways of these DEGs, we conducted functional enrichment analysis. Our GO results displayed that these genes are mainly involved in response to nutrient levels (GO: 0031667), cellular response to chemical stress (GO: 0062197) and response to oxidative stress (GO: 0034599) in biological processes. As for cell components, these genes are mainly enriched in organelle outer membrane (GO: 0031968), outer membrane (GO: 0019867) and mitochondrial outer membrane (GO: 0005741). In terms of molecular function, these genes were mainly enriched in DNA‐binding transcription factor binding (GO: 0140297) and ubiquitin protein ligase binding (GO: 0031625) (Figure 2A,B).

The results of KEGG (Figure 2C) manifested that DEGs are mainly connected with the PI3K‐Akt signaling pathway, autophagy, and some virus infections (Human cytomegalovirus infection, Kaposi sarcoma‐associated herpesvirus infection, and viral hepatitis). GSEA consequence showed that these genes are mainly abundant in P53 signaling pathway, Pyrimidine metabolism, Ribosome, Spliceosome and Huntingtons disease. Our discoveries suggest that the cell metabolic changes may be closely related to MM (Figure 2D).

### 3.3 Results of WGCNA

**FIGURE 3:**
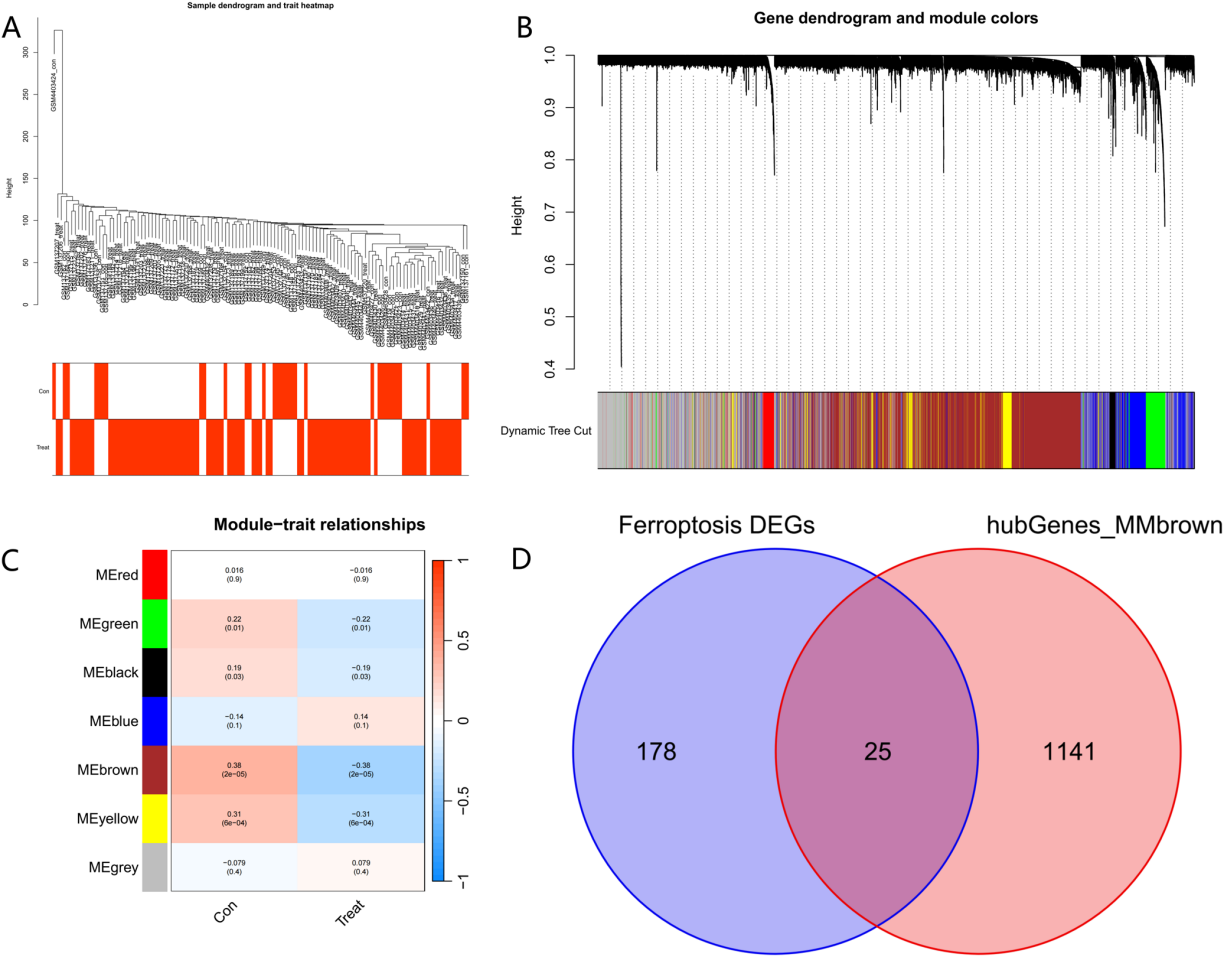
WGCNA coexpression network. (A) Sample clustering dendrogram of GSE5900 and GSE146649. (B) Dendrogram of genes clustered via the dissimilarity measure (1‐TOM). (C) Heatmap of the correlation between genes and clinical traits. (D) Venn diagram of ferroptosis DEGs and brown module.

In order to get the functional clusters in MM patients, we selected 21 654 genes from GSE5900 and GSE146649. Afterward, a sample dendrogram and trait heatmap were drawn (Figure 3A). We chose *β* = 8 (R2 = 0.86) as the appropriate soft threshold. Seven modules were identified by the TOM matrix. Similar modules on the clustering tree were merged (Figure 3B). Then, we evaluated the association among all modules and two clinical traits (tumor and normal) according to the plotted heatmap of module‐trait relationships (Figure 3C). Our research findings proved that the brown module had the closest relationship with MM (*r* = 0.38, *p* = 2e−05) and contained 3148 genes. According to the critical values: module membership value > 0.50 and Gene Significance (GS) value > 0.2, we collected a total of 1141 genes in the brown module as the hub genes. The intersection of ferroptosis‐related DEGs and WGCNA has 25 genes (Figure 3D), we considered them as “real” potential hub genes.

### 3.4 Identification of Hub Genes

**FIGURE 4:**
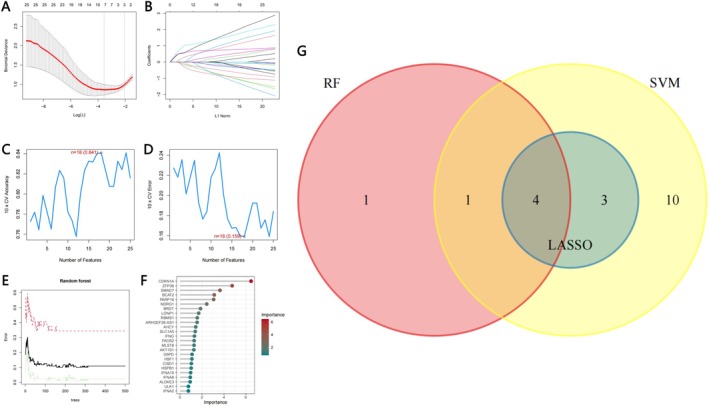
Screening hub genes by machine learning. (A, B) The variable selection in LASSO regression (*n* = 7). (C, D) Optimal biomarkers screening by SVM algorithm (*n* = 18). (E, F) Significant genes selected via the random forest method (*n* = 7). (G) Venn of overlapping genes in three algorithms.

We extracted seven potential hub genes (SMAD7, IFNA2, ARHGEF26‐AS1, CDKN1A, ZFP36, BCAT2, BRDT) after LASSO regression analysis (Figure 4A,B). The classifier error was minimized when the number of features was 18 in the SVM (Figure 4C,D). CDKN1A, BCAT2, ALOXE3, IFNA2, MLST8, ARHGEF26‐AS1, BRDT, ZFP36, HSF1, CISD1, ULK1, AHCY, SMAD7, IFNA10, NDRG1, AKT1S1, FADS2, and HSPB1 were screened as hub biomarkers. Random Forest analysis identified six genes with importance scores > 2.0 as potential diagnostic features for MM: CDKN1A, ZFP36, SMAD7, BCAT2, PARP16, and NDRG1 (Figure 4E,F). Four overlapping genes (SMAD7, CDKN1A, ZFP36, BCAT2) identified across these three methodologies were ultimately designated as candidate biomarkers (Figure 4G).

### 3.5 Reliability Evaluation of Hub Genes

**FIGURE 5:**
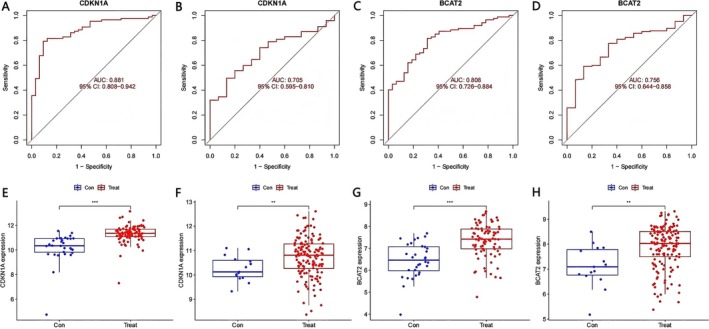
Effectiveness evaluation of *CDKN1A* and *BCAT2* (***p < 0.001, **p < 0.01, *p < 0.05, “ns”: *p* > 0.05) (A) ROC of *CDKN1A* in test set. (B) ROC of *CDKN1A* in validation set. (C) ROC of *BCAT2* in test set. (D) ROC of *BCAT2* in validation set. (E) Boxplot of *CDKN1A* in test set. (F) Boxplot of *CDKN1A* in validation set. (G) Boxplot of *BCAT2* in test set. (H) Boxplot of *BCAT2* in validation set.

We used ROC curves and drew boxplots to verify the reliability of our overlapping hub genes. After that, we removed several genes that had inconsistent results in the test set and validation set (GSE6477), and selected the two genes (CDKN1A, BCAT2) with the highest reliability. In ROC curves, the area under the curve (AUC) values for CDKN1A were 0.881 (Figure 5A) in the test set and 0.705 in the validation set (Figure 5B). The AUC values for BCAT2 were 0.808 (Figure 5C) and 0.756 (Figure 5D).

For the boxplots, whether in the test set or validation set, we are able to see that CDKN1A and BCAT2 are highly expressed in the treatment group, and their *p*‐value is also less than 0.01 (Figure 5E–H). This indicates that the expression level of CDKN1A and BCAT2 in the validation set is congruent with the expression level in the test set.

### 3.6 BCAT2 and CDKN1A Verification in MM Cell Lines

**FIGURE 6:**
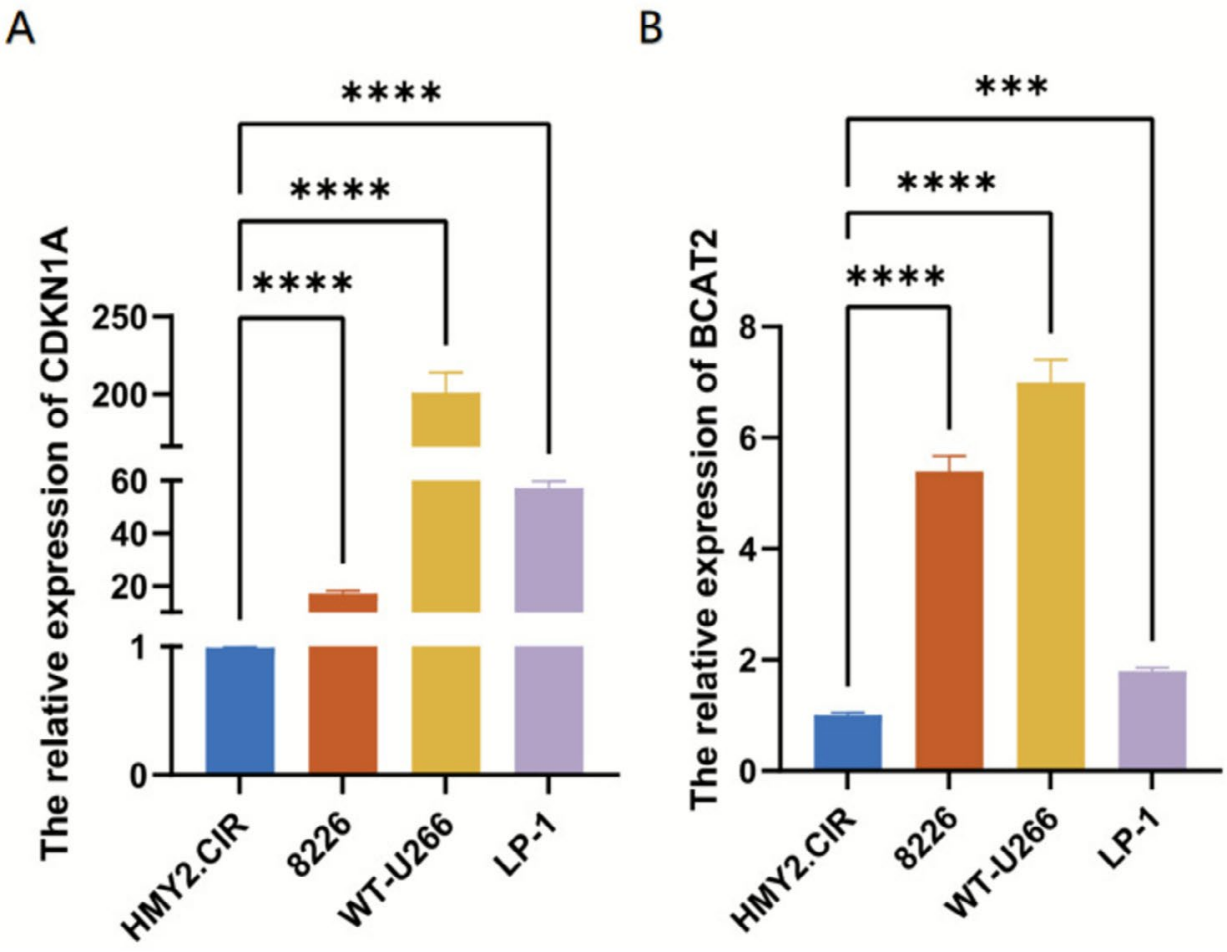
The mRNA expression validation of *CDKN1A* and *BCAT2* between control and MM cells by RT‐qPCR (****p < 0.0001, ***p < 0.001, **p < 0.01, *p < 0.05, “ns”: *p* > 0.05). (A) The relative expression of *CDKN1A* in RPMI 8226, WT‐U266 and LP‐1. (B) The relative expression of *BCAT2* in RPMI 8226, WT‐U266 and LP‐1.

To verify the mRNA expression of these two hub genes (CDKN1A and BCAT2), three MM cell lines of RPMI 8226, WT‐U266, LP‐1 and HMy2.CIR cells were cultured. RT‐qPCR results showed the mRNA expression levels of CDKN1A in MM RPMI 8226, WT‐U266 and LP‐1 cell lines were significantly higher than those in the control HMy2.CIR cell line (*p* < 0.05, Figure 6A). At the same time, we found that the mRNA expression of BCAT2 was also overexpression in these three MM cell lines (*p* < 0.05, Figure 6B).

### 3.7 Serum BCAT2 and CDKN1A Verification in MM Cohort

**FIGURE 7:**
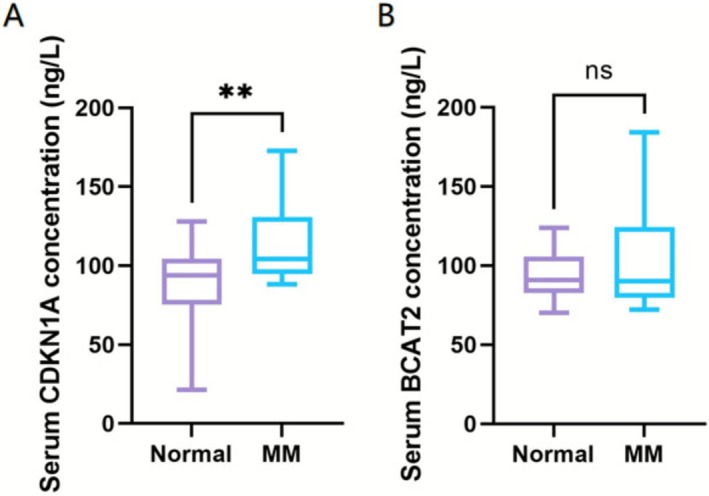
Serum CDKN1A and BCAT1 verification between normal controls and MM patients via ELISA (**p < 0.01, *p < 0.05, “ns”: *p* > 0.05). (A) Serum CDKN1A concentration in normal controls and MM patients (ng/L). (B) Serum BCAT2 concentration in normal controls and MM patients (ng/L).

To further validate protein expression of the hub genes (CDKN1A and BCAT2), serum samples were collected from 30 healthy controls and 30 newly diagnosed MM patients. ELISA quantification revealed significantly elevated relative expression of CDKN1A protein in the MM cohort compared to healthy controls (*p <* 0.01, Figure 7A), the result showing concordance with upregulated CDKN1A mRNA levels detected by RT‐qPCR in MM cells. However, the protein expression of BCAT2 exhibited comparable levels between MM patients and controls (*p* > 0.05, Figure 7B).

## 4 Discussion

As a malignant plasma cell disease, MM can lead to osteolytic lesions, renal insufficiency, and hypercalcemia, which will make patients more susceptible to being infected [25]. Despite the continuous efforts of researchers, MM remains an incurable malignant tumor [26].

In recent times, the treatments of MM have gradually developed into specific targeted therapies as people's understanding of it becomes deepened [27]. Since scientists discovered ferroptosis, it has opened up new horizons for the research of pathogenesis, but related research about MM still requires further refinement [28]. Therefore, screening for FRGs and regulatory pathways associated with MM may provide new ideas for diagnosis and treatment.

We acquired 178 DEGs after conducting differential expression analysis of FRGs in GSE5900 and GSE146649. GO functional enrichment analysis indicated our 178 substantially expressed FRGs in MM are mostly abundant in biological processes such as nutrient levels, oxidative stress, and chemical stress. In various hematological malignancies, oxidative stress is an important factor that induces ferroptosis. For example, glutathione peroxidase 4 (GPX4) was overexpressed in various hematologic malignancies, including acute myeloid leukemia (AML), where GPX family members were upregulated under oxidative stress and correlated with clinical prognosis [29]. In AML, high mobility group box 1 (HMGB1) modulated the RAS‐JNK/p38 pathway by altering lipid reactive oxygen species (ROS) production, thereby influencing oxidative stress and inducing ferroptosis [30]. A ferroptosis‐related prognostic model incorporating GPX4 demonstrated robust prognostic utility in diffuse large B‐cell lymphoma (DLBCL) [31]. Current evidence indicates that while ferroptosis in hematologic malignancies predominantly involves oxidative stress, substantial mechanistic heterogeneity exists across distinct contexts.

Recent studies have suggested that parameters related to oxidative stress may help identify potential therapeutic targets for MM [32] and develop strategies to control disease progression [33]. Ferroptosis triggered by reactive ROS helps to inhibit tumor growth and enhance chemosensitivity [34]. Similar to AML, GPX4 expression was elevated in MM plasma cells compared to healthy counterparts, suggesting potential utilization of this antioxidant pathway to counteract elevated ROS levels [35]. Inhibition of glutamate‐cysteine ligase, the first enzyme in glutathione (GSH) biosynthesis, reduced GSH levels in MM cells and enhanced bortezomib efficacy [36], demonstrating that GSH‐ROS interplay critically regulates ferroptosis in MM. We speculate the oxidative stress‐induced ferroptosis should also be applicable to the potential therapeutic targets of multiple myeloma.

KEGG pathway enrichment analysis showed us these DEGs are primarily concentrated on PI3K/Akt pathways and autophagy. The results of GSEA showed that these 178 genes are mainly enriched in the P53 signaling pathway, pyrimidine metabolism, ribosome, and spliceosome. Apigenin can inhibit the proliferation of MM cells by inducing ferroptosis and autophagy through downregulating signal transducer and activator of transcription 1 and AKT [37]. As mentioned before, GPX4 inhibitor (RSL3 or ML162) was used in combination with bortezomib or lenalidomide in RPMI‐8226 MM cells, synergistically reducing the proliferation of MM cells and inducing ferroptosis [38]. We speculate that there might be metabolic signals and autophagy abnormalities in MM, as well as anomalies in transcription and translation.

In the subsequent WGCNA analysis, the brown module containing 1141 hub genes showed the strongest correlation with MM. Use a Venn diagram to filter out the genes which are related to ferroptosis as the intersection genes. We screened two genes as biomarkers of MM (CDKN1A, BCAT2) by LASSO regression, SVM, and Random Forest.

We charted the ROC curves of CDKN1A and BCAT2 for bioinformatics validation, and their AUC values in the test set and the validation set were both greater than 0.7. This indicates that CDKN1A and BCAT2 possess significant diagnostic value and may come to be ferroptosis‐related biomarkers of MM.

RT‐qPCR was employed to validate the mRNA expression of the hub genes in MM cells. The results showed the relative expression levels of CDKN1A and BCAT2 are all upregulated in three types of cells, which cohere with the data analysis findings. Meanwhile, ELISA results also revealed elevated CDKN1A protein levels in MM patient serum, while BCAT2 showed no significant alteration. Future studies expanding the patient cohort cases or incorporating bone marrow examinations for further validation might yield divergent outcomes.

Cyclin dependent kinase inhibitor 1A (CDKN1A) encodes for p21, is considered a type of cyclin‐dependent kinase inhibitor; its primary function is to impede cell cycle progression [39]. However, many tumors, including primary hepatocellular carcinoma [40] show elevated CDKN1A expression, which is associated with high malignancy and poor prognosis [41]. CDKN1A may exhibit both oncogenic and tumor‐suppressive roles in the progression of cancer.

Xudong Zhang et al. found that CDKN1A is highly expressed in high‐grade gliomas compared to normal brain tissue in 2022 [42]. Tuerxun N et al. also discovered that CDKN1A is highly expressed in their study of key genes regulating the immune microenvironment in multiple myeloma bone marrow [43]. Their results align with our findings. Besides, p21 can promote the binding of cyclin D with CDK4 or CDK6 complexes without inhibiting their kinase activity, thereby facilitating tumorigenesis independently of its anti‐apoptotic activity [44], which may be one of its carcinogenic mechanisms.

Reasons for high expression of CDKN1A in certain tumors remain unclear. But high expression of CDKN1A can cause cell cycle arrest, allowing malignant cells to get sufficient time to repair genetic damage [45]. And this might exhibit an anti‐apoptotic effect in regulating apoptosis signaling pathways. The cytostatic effect of p21 and its concomitant suppression of apoptosis can be counterbalanced through multiple molecular mechanisms, including selective transcriptional repression of CDKN1A [46] and activation of pro‐apoptotic genes [47]. Another recent finding collectively demonstrates that upregulated YTHDF2 in malignant plasma cells promotes MM proliferation through the EGR1/p21 regulatory axis [48]. A study showed that dihydroartemisinin could induce cell cycle arrest in MM cells, and the cell cycle arrest is closely related to the production of ROS [49], suggesting that CDKN1A may also play a similar role. This study only preliminarily identified the expression of *CDKN1A* in the 8226, WT‐U266, and LP‐1 MM cell lines is rising, indicating high *CDKN1A* has a connection with the occurrence and development of MM. Still, the exact operational mechanisms need more investigation.

*BCAT2* is a protein code gene that encodes branched‐chain amino acid (BCAA) transaminase 2. This gene encodes a dimeric protein that functions to facilitate the first step in the metabolism of the BCAAs leucine, isoleucine, or valine [50]. It is commonly believed that mutations in BCAT2 are associated with hypervalinemia and hyperleucinemia [51].

Some research has demonstrated that BCAT2 expressed aberrantly in multiple kinds of neoplasms [52]. Jintao Li et al. found that pancreatic tissue‐specific knockout of BCAT2 impedes development of pancreatic intraepithelial neoplasia (PanIN). And the expression of BCAT2 is enhanced in mouse models and human pancreatic ductal adenocarcinoma (PDAC) [53]. This indicated *BCAT2*‐mediated BCAA deamination is crucial for the occurrence of PDAC. In prostate cancer, BCAT2 physically interacted with PCBP1 at position leucine 239 through hydrogen‐bond networking, and their complex cooperatively activated the PI3K/AKT signaling pathway [54]. PCBP1 is an RNA‐binding protein, overexpression of PCBP1 suppressed LC3B expression, resulting in reduced autophagy [55]. In this study, KEGG pathway enrichment analysis also revealed significant enrichment of DEGs in the PI3K‐Akt signaling pathway, which may be mechanistically linked to BCAT2 activity.

Recent studies have identified some compounds that induce ferroptosis in hematologic malignancies by targeting GPX4, lipid ROS, and system xc‐. Given that CDKN1A‐mediated cell cycle arrest induces ROS dysregulation, and BCAT2 functions as a mitochondrial isoform involved in respiratory metabolism [56].

In this study, multiple innovative bioinformatics techniques were employed to screen ferroptosis‐related DEGs (CDKN1A and BCAT2) of MM, and RT‐PCR and ELISA experiments were conducted for validation in MM cells and the MM cohort. However, there are still limitations to this study. For example, we lack analysis of the correlation between the expression levels of CDKN1A and BCAT2 genes and other laboratory parameters in MM patients, and research on how these factors impact the prognosis and survival of MM patients. Moreover, there is a lack of a deeper delve into the molecular mechanisms by which CDKN1A and BCAT2 genes link ferroptosis to MM progression and resistance to therapies.

In future research, we will further investigate the specific biological roles of CDKN1A and BCAT2 genes in MM at the clinical cohorts, cellular, and animal experiments. In clinical cohorts, the correlations between high or low expression levels of CDKN1A and BCAT2 genes and other laboratory parameters including serum iron and ferritin concentration, and disease progression or treatment resistance of MM patients were observed through a long‐term follow‐up study. In vitro experiments, knock out or overexpress CDKN1A and BCAT2 were conducted to construct lentiviral vectors, which were transduced into MM cell lines. The biological effects of these vectors on cell proliferation, invasion, migration, cell cycle distribution, and apoptosis were systematically evaluated, and the dynamic changes of Fe^2+^/ROS in cells were observed. In vivo experiments, a xenograft MM mouse model was established using transfected knockout or overexpressing CDKN1A and BCAT2 cell lines, for analyzing the effects of CDKN1A or BCAT2 genes on tumor growth and MM cell autophagy. By integrating evidence from clinical cohorts, cellular, and animal experiments, our previous bioinformatics research could be comprehensively validated to enhance the robustness and reliability of the research findings.

## 5 Conclusions

Overall, our research findings reveal that CDKN1A and BCAT2 have the potential to become ferroptosis‐related biomarkers in MM and are also closely related to various malignancies. These results have been further validated in RPMI 8226, WT‐U266, LP‐1 MM cell lines, and partially in the serum samples of MM patients. This study demonstrates ferroptosis‐associated dysregulated targets in MM, providing novel therapeutic perspectives.

## Author Contributions

**Yifan Wang:** software (lead), validation (lead), writing – original draft (lead). **Xing Xie:** methodology (lead), software (supporting). **Mengyuan Gu:** validation (equal). **Yanting Zheng:** investigation (supporting). **Jing Wu:** resources (supporting). **Qicai Wang:** data curation (supporting). **Zhian Ling:** conceptualization (equal). **Ruolin Li:** conceptualization (equal), funding acquisition (supporting), project administration (lead), writing – review and editing (lead).

## Funding

This work was supported by Guangxi Province Health Technology Development and Application Project, No. S2018076.

## Ethics Statement

The study involving human samples was approved by the Research Ethics Committee of the First Affiliated Hospital of Guangxi Medical University (No: 2025‐E0347). All procedures complied with local legislation and institutional regulations, and all participants signed informed consent forms.

## Conflicts of Interest

The authors declare no conflicts of interest.

## Data Availability

The data presented in this study are openly available in Gene Expression Omnibus (https://www.ncbi.nlm.nih.gov/geo/), reference number [GSE5900, GSE6477 and GSE146649].
